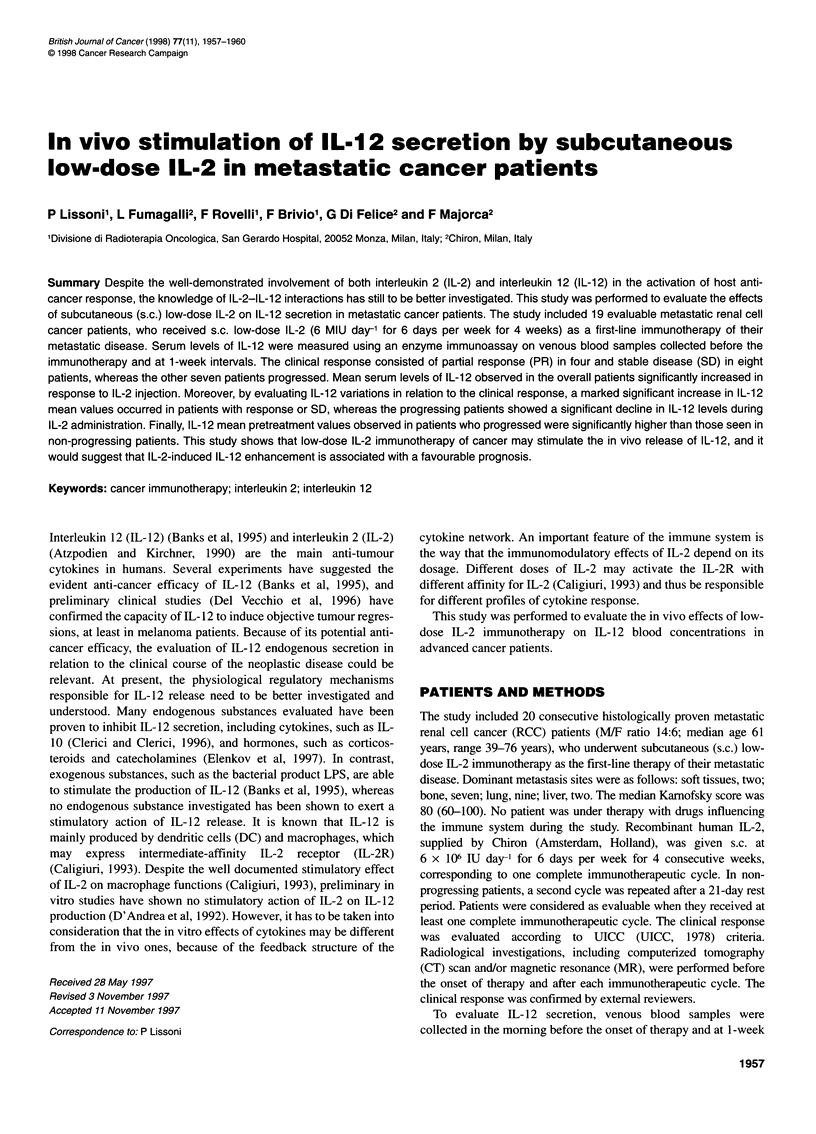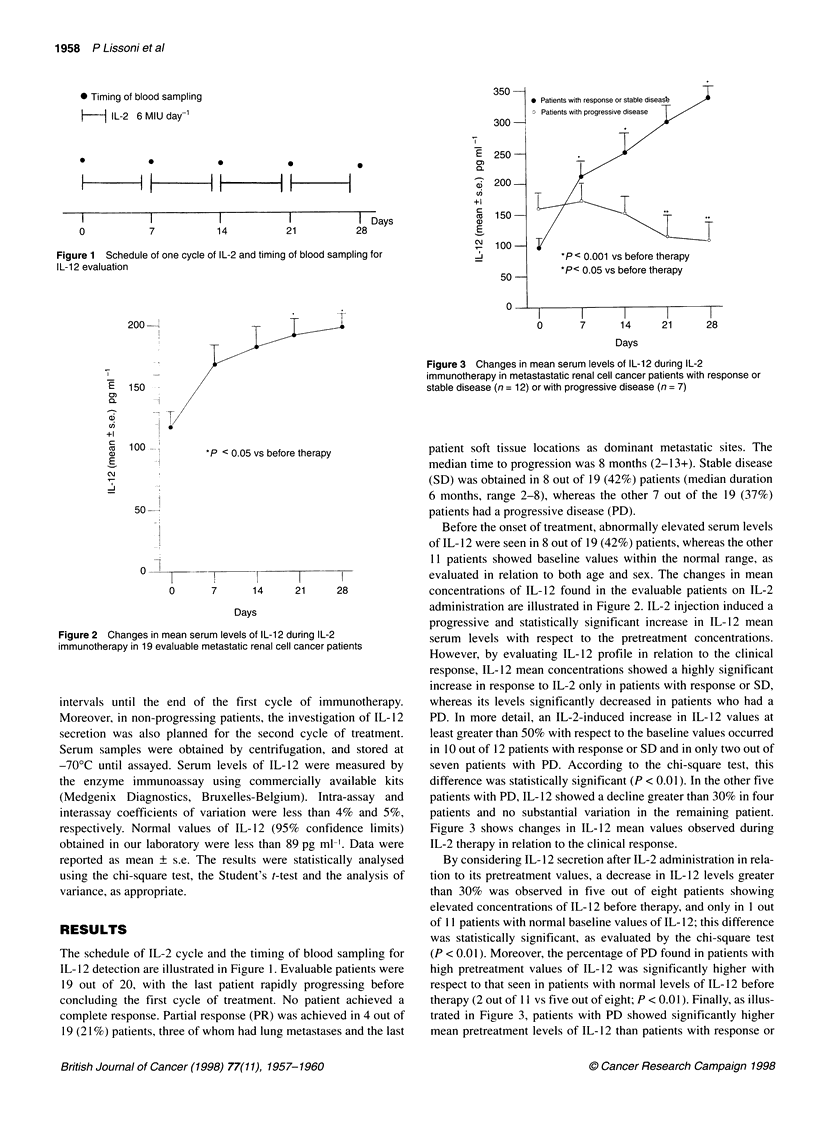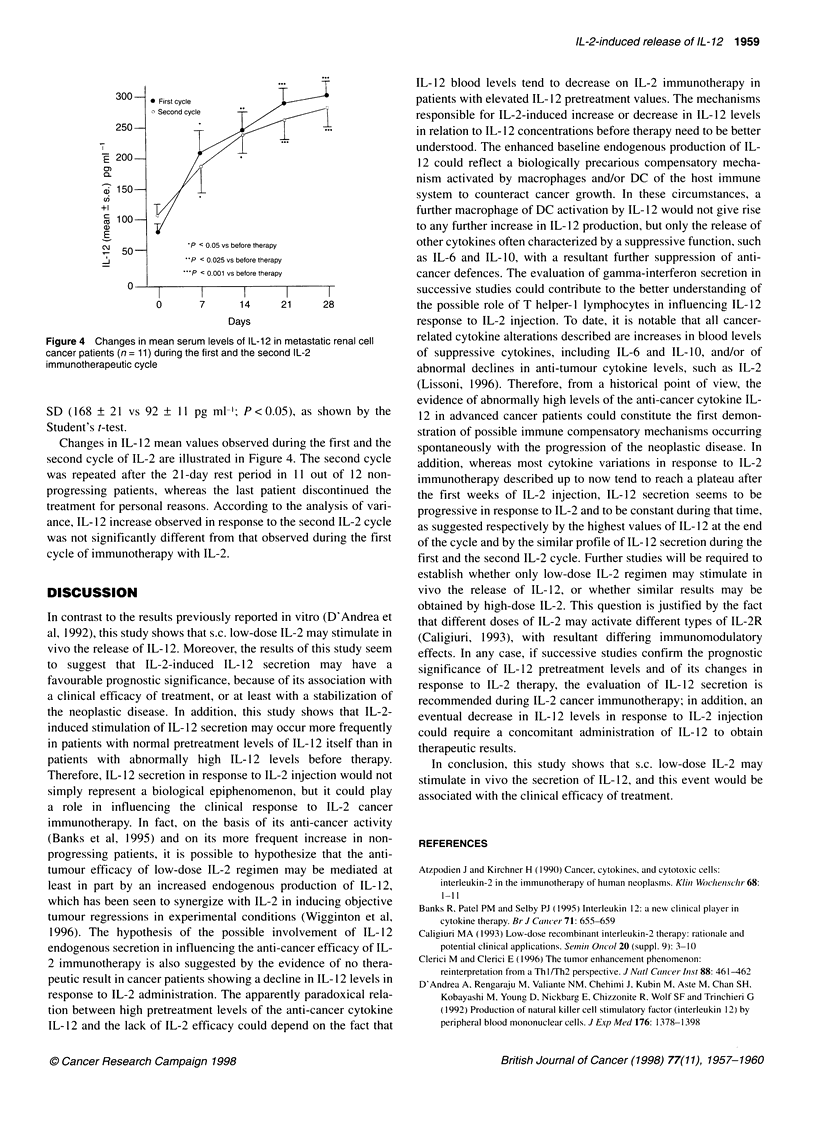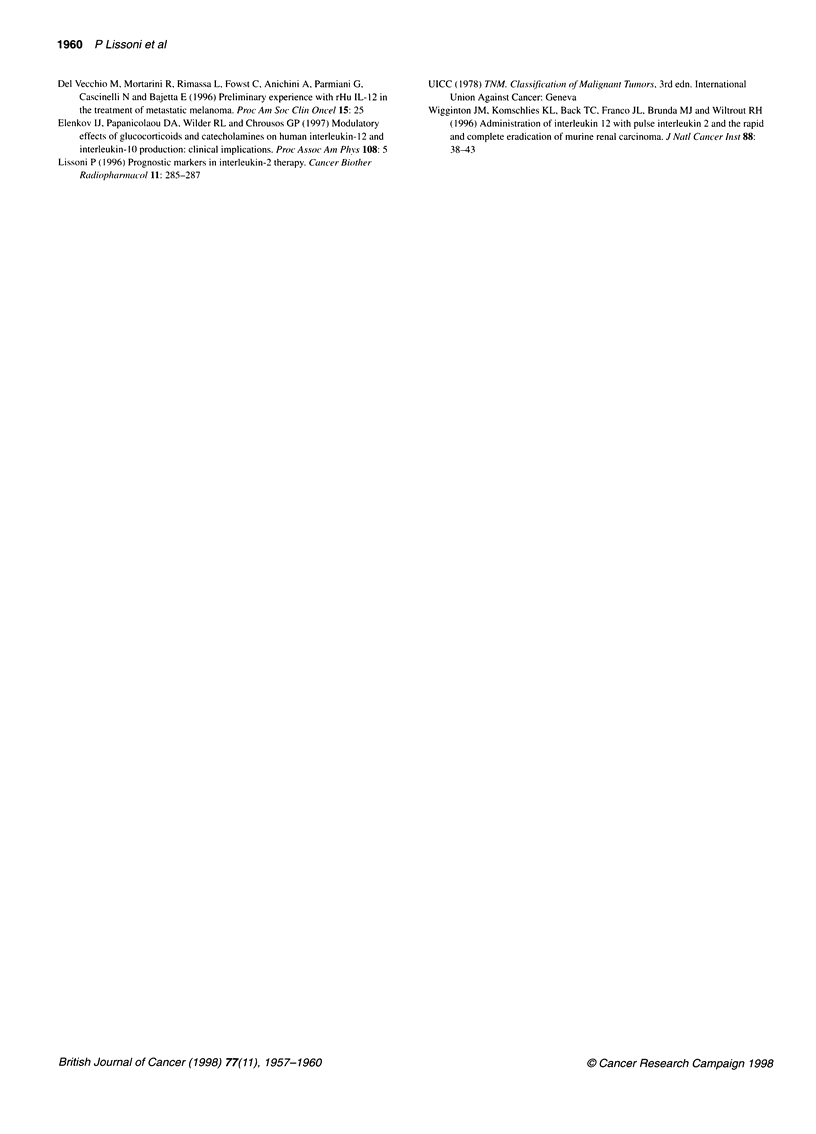# In vivo stimulation of IL-12 secretion by subcutaneous low-dose IL-2 in metastatic cancer patients.

**DOI:** 10.1038/bjc.1998.324

**Published:** 1998-06

**Authors:** P. Lissoni, L. Fumagalli, F. Rovelli, F. Brivio, G. Di Felice, F. Majorca

**Affiliations:** Divisione di Radioterapia Oncologica, San Gerardo Hospital, Milan, Italy.

## Abstract

Despite the well-demonstrated involvement of both interleukin 2 (IL-2) and interleukin 12 (IL-12) in the activation of host anti-cancer response, the knowledge of IL-2-IL-12 interactions has still to be better investigated. This study was performed to evaluate the effects of subcutaneous (s.c.) low-dose IL-2 on IL-12 secretion in metastatic cancer patients. The study included 19 evaluable metastatic renal cell cancer patients, who received s.c. low-dose IL-2 (6 MIU day(-1) for 6 days per week for 4 weeks) as a first-line immunotherapy of their metastatic disease. Serum levels of IL-12 were measured using an enzyme immunoassay on venous blood samples collected before the immunotherapy and at 1-week intervals. The clinical response consisted of partial response (PR) in four and stable disease (SD) in eight patients, whereas the other seven patients progressed. Mean serum levels of IL-12 observed in the overall patients significantly increased in response to IL-2 injection. Moreover, by evaluating IL-12 variations in relation to the clinical response, a marked significant increase in IL-12 mean values occurred in patients with response or SD, whereas the progressing patients showed a significant decline in IL-12 levels during IL-2 administration. Finally, IL-12 mean pretreatment values observed in patients who progressed were significantly higher than those seen in non-progressing patients. This study shows that low-dose IL-2 immunotherapy of cancer may stimulate the in vivo release of IL-12, and it would suggest that IL-2-induced IL-12 enhancement is associated with a favourable prognosis.


British Journal of Cancer (1998) 77(11), 1957–1960
© 1998 Cancer Research Campaign

### In vivo stimulation of IL-12 secretion by subcutaneous low-dose IL-2 in metastatic cancer patients

**P Lissoni[1], L Fumagalli[2], F Rovelli[1], F Brivio[1], G Di Felice[2] and F Majorca[2]**

[1]Divisione di Radioterapia Oncologica, San Gerardo Hospital, 20052 Monza, Milan, Italy; [2]Chiron, Milan, Italy

**Summary** Despite the well-demonstrated involvement of both interleukin 2 (IL-2) and interleukin 12 (IL-12) in the activation of host anti-cancer response, the knowledge of IL-2–IL-12 interactions has still to be better investigated. This study was performed to evaluate the effects of subcutaneous (s.c.) low-dose IL-2 on IL-12 secretion in metastatic cancer patients. The study included 19 evaluable metastatic renal cell cancer patients, who received s.c. low-dose IL-2 (6 MIU day$^{-1}$ for 6 days per week for 4 weeks) as a first-line immunotherapy of their metastatic disease. Serum levels of IL-12 were measured using an enzyme immunoassay on venous blood samples collected before the immunotherapy and at 1-week intervals. The clinical response consisted of partial response (PR) in four and stable disease (SD) in eight patients, whereas the other seven patients progressed. Mean serum levels of IL-12 observed in the overall patients significantly increased in response to IL-2 injection. Moreover, by evaluating IL-12 variations in relation to the clinical response, a marked significant increase in IL-12 mean values occurred in patients with response or SD, whereas the progressing patients showed a significant decline in IL-12 levels during IL-2 administration. Finally, IL-12 mean pretreatment values observed in patients who progressed were significantly higher than those seen in non-progressing patients. This study shows that low-dose IL-2 immunotherapy of cancer may stimulate the in vivo release of IL-12, and it would suggest that IL-2-induced IL-12 enhancement is associated with a favourable prognosis.

**Keywords:** cancer immunotherapy; interleukin 2; interleukin 12

Interleukin 12 (IL-12) (Banks et al, 1995) and interleukin 2 (IL-2) (Atzpodien and Kirchner, 1990) are the main anti-tumour cytokines in humans. Several experiments have suggested the evident anti-cancer efficacy of IL-12 (Banks et al, 1995), and preliminary clinical studies (Del Vecchio et al, 1996) have confirmed the capacity of IL-12 to induce objective tumour regressions, at least in melanoma patients. Because of its potential anti-cancer efficacy, the evaluation of IL-12 endogenous secretion in relation to the clinical course of the neoplastic disease could be relevant. At present, the physiological regulatory mechanisms responsible for IL-12 release need to be better investigated and understood. Many endogenous substances evaluated have been proven to inhibit IL-12 secretion, including cytokines, such as IL-10 (Clerici and Clerici, 1996), and hormones, such as corticosteroids and catecholamines (Elenkov et al, 1997). In contrast, exogenous substances, such as the bacterial product LPS, are able to stimulate the production of IL-12 (Banks et al, 1995), whereas no endogenous substance investigated has been shown to exert a stimulatory action of IL-12 release. It is known that IL-12 is mainly produced by dendritic cells (DC) and macrophages, which may express intermediate-affinity IL-2 receptor (IL-2R) (Caligiuri, 1993). Despite the well documented stimulatory effect of IL-2 on macrophage functions (Caligiuri, 1993), preliminary in vitro studies have shown no stimulatory action of IL-2 on IL-12 production (D'Andrea et al, 1992). However, it has to be taken into consideration that the in vitro effects of cytokines may be different from the in vivo ones, because of the feedback structure of the cytokine network. An important feature of the immune system is the way that the immunomodulatory effects of IL-2 depend on its dosage. Different doses of IL-2 may activate the IL-2R with different affinity for IL-2 (Caligiuri, 1993) and thus be responsible for different profiles of cytokine response.

This study was performed to evaluate the in vivo effects of low-dose IL-2 immunotherapy on IL-12 blood concentrations in advanced cancer patients.

*Received 28 May 1997*
*Revised 3 November 1997*
*Accepted 11 November 1997*

*Correspondence to:* P Lissoni

### PATIENTS AND METHODS

The study included 20 consecutive histologically proven metastatic renal cell cancer (RCC) patients (M/F ratio 14:6; median age 61 years, range 39–76 years), who underwent subcutaneous (s.c.) low-dose IL-2 immunotherapy as the first-line therapy of their metastatic disease. Dominant metastasis sites were as follows: soft tissues, two; bone, seven; lung, nine; liver, two. The median Karnofsky score was 80 (60–100). No patient was under therapy with drugs influencing the immune system during the study. Recombinant human IL-2, supplied by Chiron (Amsterdam, Holland), was given s.c. at $6 \times 10^6$ IU day$^{-1}$ for 6 days per week for 4 consecutive weeks, corresponding to one complete immunotherapeutic cycle. In non-progressing patients, a second cycle was repeated after a 21-day rest period. Patients were considered as evaluable when they received at least one complete immunotherapeutic cycle. The clinical response was evaluated according to UICC (UICC, 1978) criteria. Radiological investigations, including computerized tomography (CT) scan and/or magnetic resonance (MR), were performed before the onset of therapy and after each immunotherapeutic cycle. The clinical response was confirmed by external reviewers.

To evaluate IL-12 secretion, venous blood samples were collected in the morning before the onset of therapy and at 1-week

**Figure 1** Schedule of one cycle of IL-2 and timing of blood sampling for IL-12 evaluation

**Figure 2** Changes in mean serum levels of IL-12 during IL-2 immunotherapy in 19 evaluable metastatic renal cell cancer patients

**Figure 3** Changes in mean serum levels of IL-12 during IL-2 immunotherapy in metastastatic renal cell cancer patients with response or stable disease ($n$ = 12) or with progressive disease ($n$ = 7)

intervals until the end of the first cycle of immunotherapy. Moreover, in non-progressing patients, the investigation of IL-12 secretion was also planned for the second cycle of treatment. Serum samples were obtained by centrifugation, and stored at −70°C until assayed. Serum levels of IL-12 were measured by the enzyme immunoassay using commercially available kits (Medgenix Diagnostics, Bruxelles-Belgium). Intra-assay and interassay coefficients of variation were less than 4% and 5%, respectively. Normal values of IL-12 (95% confidence limits) obtained in our laboratory were less than 89 pg $ml^{-1}$. Data were reported as mean ± s.e. The results were statistically analysed using the chi-square test, the Student's $t$-test and the analysis of variance, as appropriate.

### RESULTS

The schedule of IL-2 cycle and the timing of blood sampling for IL-12 detection are illustrated in Figure 1. Evaluable patients were 19 out of 20, with the last patient rapidly progressing before concluding the first cycle of treatment. No patient achieved a complete response. Partial response (PR) was achieved in 4 out of 19 (21%) patients, three of whom had lung metastases and the last patient soft tissue locations as dominant metastatic sites. The median time to progression was 8 months (2–13+). Stable disease (SD) was obtained in 8 out of 19 (42%) patients (median duration 6 months, range 2–8), whereas the other 7 out of the 19 (37%) patients had a progressive disease (PD).

Before the onset of treatment, abnormally elevated serum levels of IL-12 were seen in 8 out of 19 (42%) patients, whereas the other 11 patients showed baseline values within the normal range, as evaluated in relation to both age and sex. The changes in mean concentrations of IL-12 found in the evaluable patients on IL-2 administration are illustrated in Figure 2. IL-2 injection induced a progressive and statistically significant increase in IL-12 mean serum levels with respect to the pretreatment concentrations. However, by evaluating IL-12 profile in relation to the clinical response, IL-12 mean concentrations showed a highly significant increase in response to IL-2 only in patients with response or SD, whereas its levels significantly decreased in patients who had a PD. In more detail, an IL-2-induced increase in IL-12 values at least greater than 50% with respect to the baseline values occurred in 10 out of 12 patients with response or SD and in only two out of seven patients with PD. According to the chi-square test, this difference was statistically significant ($P < 0.01$). In the other five patients with PD, IL-12 showed a decline greater than 30% in four patients and no substantial variation in the remaining patient. Figure 3 shows changes in IL-12 mean values observed during IL-2 therapy in relation to the clinical response.

By considering IL-12 secretion after IL-2 administration in relation to its pretreatment values, a decrease in IL-12 levels greater than 30% was observed in five out of eight patients showing elevated concentrations of IL-12 before therapy, and only in 1 out of 11 patients with normal baseline values of IL-12; this difference was statistically significant, as evaluated by the chi-square test ($P < 0.01$). Moreover, the percentage of PD found in patients with high pretreatment values of IL-12 was significantly higher with respect to that seen in patients with normal levels of IL-12 before therapy (2 out of 11 vs five out of eight; $P < 0.01$). Finally, as illustrated in Figure 3, patients with PD showed significantly higher mean pretreatment levels of IL-12 than patients with response or

© Cancer Research Campaign 1998

**Figure 4** Changes in mean serum levels of IL-12 in metastatic renal cell cancer patients ($n$ = 11) during the first and the second IL-2 immunotherapeutic cycle

SD (168 ± 21 vs 92 ± 11 pg $ml^{-1}$; $P < 0.05$), as shown by the Student's $t$-test.

Changes in IL-12 mean values observed during the first and the second cycle of IL-2 are illustrated in Figure 4. The second cycle was repeated after the 21-day rest period in 11 out of 12 non-progressing patients, whereas the last patient discontinued the treatment for personal reasons. According to the analysis of variance, IL-12 increase observed in response to the second IL-2 cycle was not significantly different from that observed during the first cycle of immunotherapy with IL-2.

### DISCUSSION

In contrast to the results previously reported in vitro (D'Andrea et al, 1992), this study shows that s.c. low-dose IL-2 may stimulate in vivo the release of IL-12. Moreover, the results of this study seem to suggest that IL-2-induced IL-12 secretion may have a favourable prognostic significance, because of its association with a clinical efficacy of treatment, or at least with a stabilization of the neoplastic disease. In addition, this study shows that IL-2-induced stimulation of IL-12 secretion may occur more frequently in patients with normal pretreatment levels of IL-12 itself than in patients with abnormally high IL-12 levels before therapy. Therefore, IL-12 secretion in response to IL-2 injection would not simply represent a biological epiphenomenon, but it could play a role in influencing the clinical response to IL-2 cancer immunotherapy. In fact, on the basis of its anti-cancer activity (Banks et al, 1995) and on its more frequent increase in non-progressing patients, it is possible to hypothesize that the anti-tumour efficacy of low-dose IL-2 regimen may be mediated at least in part by an increased endogenous production of IL-12, which has been seen to synergize with IL-2 in inducing objective tumour regressions in experimental conditions (Wigginton et al, 1996). The hypothesis of the possible involvement of IL-12 endogenous secretion in influencing the anti-cancer efficacy of IL-2 immunotherapy is also suggested by the evidence of no therapeutic result in cancer patients showing a decline in IL-12 levels in response to IL-2 administration. The apparently paradoxical relation between high pretreatment levels of the anti-cancer cytokine IL-12 and the lack of IL-2 efficacy could depend on the fact that IL-12 blood levels tend to decrease on IL-2 immunotherapy in patients with elevated IL-12 pretreatment values. The mechanisms responsible for IL-2-induced increase or decrease in IL-12 levels in relation to IL-12 concentrations before therapy need to be better understood. The enhanced baseline endogenous production of IL-12 could reflect a biologically precarious compensatory mechanism activated by macrophages and/or DC of the host immune system to counteract cancer growth. In these circumstances, a further macrophage of DC activation by IL-12 would not give rise to any further increase in IL-12 production, but only the release of other cytokines often characterized by a suppressive function, such as IL-6 and IL-10, with a resultant further suppression of anti-cancer defences. The evaluation of gamma-interferon secretion in successive studies could contribute to the better understanding of the possible role of T helper-1 lymphocytes in influencing IL-12 response to IL-2 injection. To date, it is notable that all cancer-related cytokine alterations described are increases in blood levels of suppressive cytokines, including IL-6 and IL-10, and/or of abnormal declines in anti-tumour cytokine levels, such as IL-2 (Lissoni, 1996). Therefore, from a historical point of view, the evidence of abnormally high levels of the anti-cancer cytokine IL-12 in advanced cancer patients could constitute the first demonstration of possible immune compensatory mechanisms occurring spontaneously with the progression of the neoplastic disease. In addition, whereas most cytokine variations in response to IL-2 immunotherapy described up to now tend to reach a plateau after the first weeks of IL-2 injection, IL-12 secretion seems to be progressive in response to IL-2 and to be constant during that time, as suggested respectively by the highest values of IL-12 at the end of the cycle and by the similar profile of IL-12 secretion during the first and the second IL-2 cycle. Further studies will be required to establish whether only low-dose IL-2 regimen may stimulate in vivo the release of IL-12, or whether similar results may be obtained by high-dose IL-2. This question is justified by the fact that different doses of IL-2 may activate different types of IL-2R (Caligiuri, 1993), with resultant differing immunomodulatory effects. In any case, if successive studies confirm the prognostic significance of IL-12 pretreatment levels and of its changes in response to IL-2 therapy, the evaluation of IL-12 secretion is recommended during IL-2 cancer immunotherapy; in addition, an eventual decrease in IL-12 levels in response to IL-2 injection could require a concomitant administration of IL-12 to obtain therapeutic results.

In conclusion, this study shows that s.c. low-dose IL-2 may stimulate in vivo the secretion of IL-12, and this event would be associated with the clinical efficacy of treatment.

### REFERENCES

Atzpodien J and Kirchner H (1990) Cancer, cytokines, and cytotoxic cells: interleukin-2 in the immunotherapy of human neoplasms. *Klin Wochenschr* **68**: 1–11

Banks R, Patel PM and Selby PJ (1995) Interleukin 12: a new clinical player in cytokine therapy. *Br J Cancer* **71**: 655–659

Caligiuri MA (1993) Low-dose recombinant interleukin-2 therapy: rationale and potential clinical applications. *Semin Oncol* **20** (suppl. 9): 3–10

Clerici M and Clerici E (1996) The tumor enhancement phenomenon: reinterpretation from a Th1/Th2 perspective. *J Natl Cancer Inst* **88**: 461–462

D'Andrea A, Rengaraju M, Valiante NM, Chehimi J, Kubin M, Aste M, Chan SH, Kobayashi M, Young D, Nickbarg E, Chizzonite R, Wolf SF and Trinchieri G (1992) Production of natural killer cell stimulatory factor (interleukin 12) by peripheral blood mononuclear cells. *J Exp Med* **176**: 1378–1398

© Cancer Research Campaign 1998

Del Vecchio M, Mortarini R, Rimassa L, Fowst C, Anichini A, Parmiani G, Cascinelli N and Bajetta E (1996) Preliminary experience with rHu IL-12 in the treatment of metastatic melanoma. *Proc Am Soc Clin Oncel* **15**: 25

Elenkov IJ, Papanicolaou DA, Wilder RL and Chrousos GP (1997) Modulatory effects of glucocorticoids and catecholamines on human interleukin-12 and interleukin-10 production: clinical implications. *Proc Assoc Am Phys* **108**: 5

Lissoni P (1996) Prognostic markers in interleukin-2 therapy. *Cancer Biother Radiopharmacol* **11**: 285–287

UICC (1978) *TNM. Classification of Malignant Tumors*, 3rd edn. International Union Against Cancer: Geneva

Wigginton JM, Komschlies KL, Back TC, Franco JL, Brunda MJ and Wiltrout RH (1996) Administration of interleukin 12 with pulse interleukin 2 and the rapid and complete eradication of murine renal carcinoma. *J Natl Cancer Inst* **88**: 38–43

*© Cancer Research Campaign 1998*